# The Interactive Web-Based Program MSmonitor for Self-Management and Multidisciplinary Care in Persons With Multiple Sclerosis: Quasi-Experimental Study of Short-Term Effects on Patient Empowerment

**DOI:** 10.2196/14297

**Published:** 2020-03-09

**Authors:** Peter Joseph Jongen, Gezien ter Veen, Wim Lemmens, Rogier Donders, Esther van Noort, Esther Zeinstra

**Affiliations:** 1 Department of Community and Occupational Medicine University Medical Centre Groningen University of Groningen Groningen Netherlands; 2 MS4 Research Institute Nijmegen Netherlands; 3 Zorggroep Noorderboog Meppel Netherlands; 4 Isala Hospital Meppel Netherlands; 5 Radboud University Medical Center Department for Health Evidence Nijmegen Netherlands; 6 Curavista bv Geertruidenberg Netherlands

**Keywords:** multiple sclerosis, empowerment, self-management, eHealth, internet-based intervention, internet-based communication, personal autonomy, social participation, self-efficacy

## Abstract

**Background:**

Empowerment helps persons with a chronic disease to self-manage their condition and increase their autonomy and participation. MSmonitor (Curavista bv) is an interactive Web-based program for self-management and multidisciplinary care in multiple sclerosis (MS). It includes, among others, short questionnaires on fatigue (Modified Fatigue Impact Scale-5 [MFIS-5]) and health-related quality of life (HRQoL, Leeds Multiple Sclerosis Quality of Life [LMSQoL]); long questionnaires on disabilities, perception of disabilities (Multiple Sclerosis Impact Profile), and HRQoL (Multiple Sclerosis Quality of Life-54); a Medication and Adherence Inventory and an Activity Diary. The combination MFIS-5, LMSQoL, and Medication and Adherence Inventory constitutes the Quick Scan.

**Objective:**

This study aimed to investigate the short-term effects of MSmonitor on empowerment in patients with MS.

**Methods:**

We conducted a quasi-experimental study in a general hospital. Of the 180 patients with MS, 125 were eligible, 30 used MSmonitor, and 21 participated in the study (mean age 45.4 years, SD 10.2 years). A total of 24 eligible patients who did not use MSmonitor constituted the control group (mean age 49.3 years, SD 11.4 years). At baseline and at 4 months, we assessed self-efficacy (Multiple Sclerosis Self-Efficacy Scale [MSSES]), participation and autonomy (Impact on Participation and Autonomy [IPA] questionnaire), and self-management (Partners In Health [PIH] questionnaire). Differences between time points and groups were tested with paired *t* tests and χ² tests.

**Results:**

In the MSmonitor group, follow-up values remained unchanged for MSSES control (*P*=.19), MSSES function (*P*=.62), IPA limitations (*P*=.26), IPA problems (*P*=.40), PIH recognition and management of symptoms (*P*=.52), PIH adherence to treatment (*P*=.80), and PIH coping (*P*=.73), whereas the PIH knowledge score had improved (mean 27.8, SD 1.7 vs mean 28.7, SD 2.0; *P*=.02). The overall utilization rate of the program components was 83% and that of the Quick Scan was 95%. In the control group, all outcomes had remained unchanged.

**Conclusions:**

The results suggest that for first-time users of the MSmonitor program and their health care providers, it may not be justified to expect a short-term improvement in empowerment in terms of self-efficacy, self-management, autonomy, or participation. Furthermore, a lack of effect on empowerment is not because of nonusage of the program components.

## Introduction

### Background

#### Multiple Sclerosis

Multiple sclerosis (MS) is a chronic inflammatory and degenerative disease of the central nervous system (CNS). It is the most frequent chronic CNS disease in young adulthood, and the majority of patients experience their first symptoms at the age of 20 to 40 years [1]. Intermittent or continuous disease activity results in a stepwise or slow increase in disabilities over time [1]. The disease course is largely unpredictable, as is the response to disease-modifying drug (DMD) treatment [1,2]. MS is incurable, as the effectiveness of DMD treatment is only partial and limited to the inflammatory component of the disease [1,2].

#### Patient Empowerment and Web-Based Health Services

Persons with chronic conditions such as MS depend on their own insights to manage daily activities and self-care. To make optimal choices, to evaluate the effects of their choices, and to also contribute to a preventive, personalized, and participatory health care, it is paramount for them to be *empowered* [3]. Empowerment has been defined as a process: *the mechanisms by which people, organizations and communities gain mastery over their lives* [4]. Thus, patient empowerment may be defined as the process by which patients discover and develop the inherent capacity to be responsible for one’s own life [5,6]. Although the concept of patient empowerment is still developing [6,7], empowered patients are generally considered to control their situation, have a critical attitude, and participate and perform tasks in an encouraging environment [3,8,9]. A recent systematic literature review of qualitative studies identified control, coping, knowledge, participation, support, and legitimacy as key aspects of patient empowerment [6]. Notably, interventions that aimed at improving patient empowerment have resulted in higher self-efficacy and self-care competence [10].

Web-based health services use telecommunications and information technology to provide care, education, and monitoring services to patients [11]. In patients with MS, Web-based health services have been shown to result in improved health care because of improved symptom management and treatment adherence [12-15]. E-communication can be defined as communication via Web-based platforms or apps [16-18], and among MS patients, e-communication has high levels of acceptance for exchanging information with health care providers [19]. Information systems with an e-communication function have also been found to be useful in enhancing interdisciplinary communication [20,21]. Consequently, it has been suggested that e-communication should be integrated into electronic health services for patients with MS [19].

#### MSmonitor

Against this background, we developed MSmonitor, an interactive Web-based program for self-management and multidisciplinary care in persons with MS, that can be used on computers, tablets, and smart phones [22-24]. At the time of the study, MSmonitor included short questionnaires on fatigue (Modified Fatigue Impact Scale-5 [MFIS-5]) [25,26], health-related quality of life (HRQoL, Leeds Multiple Sclerosis Quality of Life [LMSQoL] questionnaire) [27,28], and anxiety and depression (Hospital Anxiety and Depression Scale [HADS]) [29-31]; long questionnaires on disabilities and perception of disabilities (Multiple Sclerosis Impact Profile [MSIP]) [32,33] and HRQoL (Multiple Sclerosis Quality of Life-54 [MSQoL-54] questionnaire) [34]; inventories (Medication and Adherence Inventory, Miction Inventory); and diaries (Activity Diary, Miction Diary) [22-24]. We previously reported that patients who used the combined MFIS-5 and LMSQoL questionnaires at least twice in a period of up to 6 months showed an improved HRQoL and that in these patients, the degree of fatigue improvement correlated with the frequency of Activity Diary usage [24].

Conceivably, MSmonitor usage may lead in various ways to an improvement of empowerment. For example, the Activity Diary and MFIS-5 give insight into factors affecting fatigue and thus facilitate self-management of fatigue and fatigue-related symptoms. The quantified overview of (perceived) disabilities given by the MSIP informs patients about the relative importance of their symptoms and thus facilitates focused self-management. Documentation of missed doses in the Medication and Adherence Inventory may help improve adherence to DMD treatment.

While developing MSmonitor, we did not intend the program to generate short-term effects, as MS is a progressive disease that most patients are afflicted with for decades. However, as we live in an *instant gratification era*, where everything seems to be available immediately via smart phone or the internet [35,36], we became aware that patients might indeed expect early results. Therefore, to obtain knowledge about the short-term effect of MSmonitor on patient empowerment, we conducted a quasi-experimental study.

### Objective

The aim of the study was to explore short-term changes in empowerment in persons with MS using MSmonitor.

## Methods

### MSmonitor

MSmonitor is used by about 1500 patients and their health care providers in 23 hospitals in the Netherlands.

#### Concept

The concept is based on the autonomy of patients, the multidisciplinary character of MS care, and the collaboration between stakeholders involved in MS care [23,24]. The program was developed gradually by the immaterial and material input of various stakeholders [22-24]. By facilitating self-assessments and self-management, MSmonitor aims to use and increase patients’ autonomy. Patients own their personal data generated by the program and decide which health care providers can have access to their data [23,24]. By making self-assessment outcomes available to the multidisciplinary team, MSmonitor helps patients in preventing unnecessary measurements and promotes the use of patient-reported outcomes [23,24].

#### Content

At the time of the study, the content comprised 9 components in 3 categories: psychometrically validated questionnaires, inventories, and diaries. The characteristics and availability of the various components are presented in Table 1.

**Figure 1:**
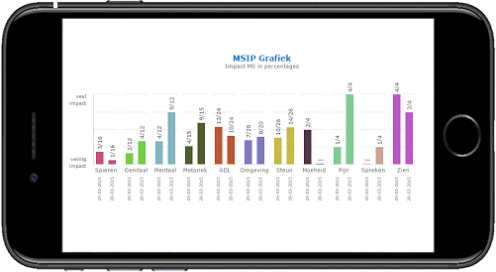
Screenshot of graphic presentation of Multiple Sclerosis Impact Profile (MSIP) disability scores in the domains muscle and movement, excretion and reproductive functions, mental functions, basic movement activities, activities of daily living, environmental factors, participation in life situations, and the symptoms fatigue, pain, speech, and vision (lower numbers). Upper numbers represent the maximum of the score range. Higher scores indicate a worse condition. Right bars give the actual score, left bars the previous score.

**Figure 2:**
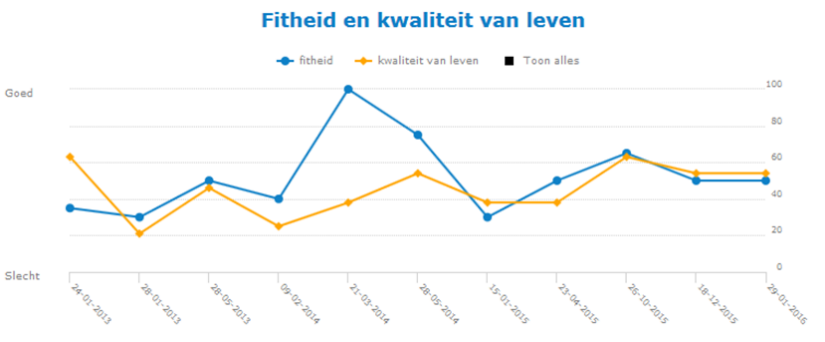
Screenshot of graphic presentation of changes over time in MFIS-5 and LMSQoL scores. The MFIS-5 score (higher is worse) is converted into a fitness (fitheid) score (higher is better) to match the direction of the LMSQoL score. Scores are converted into percentages (0%, minimum score; 100%, maximum score). MFIS-5: Modified Fatigue Impact Scale-5 items; LMSQoL: Leeds Multiple Sclerosis Quality of Life.

Alerts are sent when questionnaires are available, and reminders are sent when scheduled questionnaires are not completed. For all questionnaires, scores are automatically generated and presented in graphs and tables to patients and authorized caregivers (Figure 1), as well as changes over time (Figure 2).

**Table 1:** Characteristics and availability of MSmonitor components.

Name	Purpose	Structure	Min^a^-max^b^	Validation	Availability
MFIS-5^c^ questionnaire	Perceived impact of fatigue on daily activities over past month	5 items (0-4)	0-20 (lower=better)	Fisk et al [25]; NMSS^d^ [26]	Monthly
LMSQoL^e^ questionnaire	MS^f^-related aspects of QoL^g^ over past month	8 items (0-3)	0-24 (higher=better)	Ford et al [27]; Ensari et al [28]	Monthly
MSIP^h^ questionnaire	Overview of actual MS-related disabilities (a) and perception of disabilities (b)	36 a-items; 36 b-items; scorings variable	7 domain and 4 symptom scores, 0-variable (lower=better)	Wynia et al [32]; Wynia et al [33]	6 monthly
MSQoL^i^-54 questionnaire	Multidimensional assessment of physical and mental MS-related QoL over 4 weeks	54 items, various scorings	Physical QoL, 0-100; mental QoL, 0-100 (higher=better)	Vickrey et al [34]	Yearly
HADS^j^ questionnaire	Anxiety in past week; depression in past week	7 items (0-3); 7 items (0-3)	0-21 (lower=better); 0-21 (lower=better)	Honarmand and Feinstein [30]; Watson et al [31]	On indication
Medication and Adherence Inventory	Medication and DMD^k^ adherence in past month	N/A^l^	N/A	N/A	Monthly
Miction Inventory	Actual urological symptoms	N/A	N/A	N/A	On indication
Activity Diary	Activities and rest periods in 24 hours	N/A	N/A	N/A	Daily
Miction Diary	Frequency and quantity of miction and fluid intake in 24 hours	N/A	N/A	N/A	On indication

^a^Min: minimum score.

^b^Max: maximum score.

^c^MFIS-5: Modified Fatigue Impact Scale-5 items.

^d^NMSS: National Multiple Sclerosis Society.

^e^LMSQoL: Leeds Multiple Sclerosis Quality of Life.

^f^MS: multiple sclerosis.

^g^QoL: quality of life.

^h^MSIP: Multiple Sclerosis Impact Profile.

^i^MSQoL-54: Multiple Sclerosis Quality of Life-54 items.

^j^HADS: Hospital Anxiety and Depression Scale.

^k^DMD: disease-modifying drug.

^l^Not applicable.

Inventories provide overviews and do not generate scores; for example, the Medication and Adherence Inventory gives an update of medication that is taken, the number of missed DMD doses in the last month, and the date and reason of eventual DMD treatment discontinuation. Diaries enable the recording of specific activities or functions and thus give insight into MS-related processes over 24-hour periods [23,24]. The Activity Diary records type and duration of activities and rest periods, whereas the Miction Diary documents the frequencies and quantities of mictions and fluid intakes [23,24]. The combined use of MFIS-5, LMSQoL, and Medication and Adherence Inventory (*Quick Scan*) enables quick self-assessments of fatigue, HRQoL, and adherence to DMD treatment. The HADS, Miction Diary, and Miction Inventory are only available to patients after indication by health care professionals and were therefore not part of the study.

### Study Design

This was a prospective, quasi-experimental study. For the MSmonitor group, the baseline assessment was conducted when the participant was registered as a user. In all participants, follow-up assessment was conducted at 4 months. This follow-up period was chosen pragmatically and was dictated by the principal researcher’s availability. We considered this period justifiable, as in chronic disorders, a follow-up at 3 to 6 months is generally qualified as *short-term*.

Owing to the study’s time frame and the standard 6-month interval between consecutive completions of the MSIP and the MSQoL-54 (Table 1), the usage of these questionnaires was limited to single completions. Notably, to prevent patients from being overburdened, the program makes the MSQoL-54 available 3 months after the MSIP. Hence, 4 components were available for multiple use: the MFIS-5, LMSQoL and Medication and Adherence Inventory (Quick Scan), and the Activity Diary.

### Study Setting

The study was performed in the Neurological Department of the Isala Diaconessenhuis, Meppel, the Netherlands. The Isala Diaconessenhuis is a medium-sized (120 beds) general hospital with 1300 neurological outpatient visits per year, 5000 of which being new referrals.

### Recruitment

**Figure 3:**
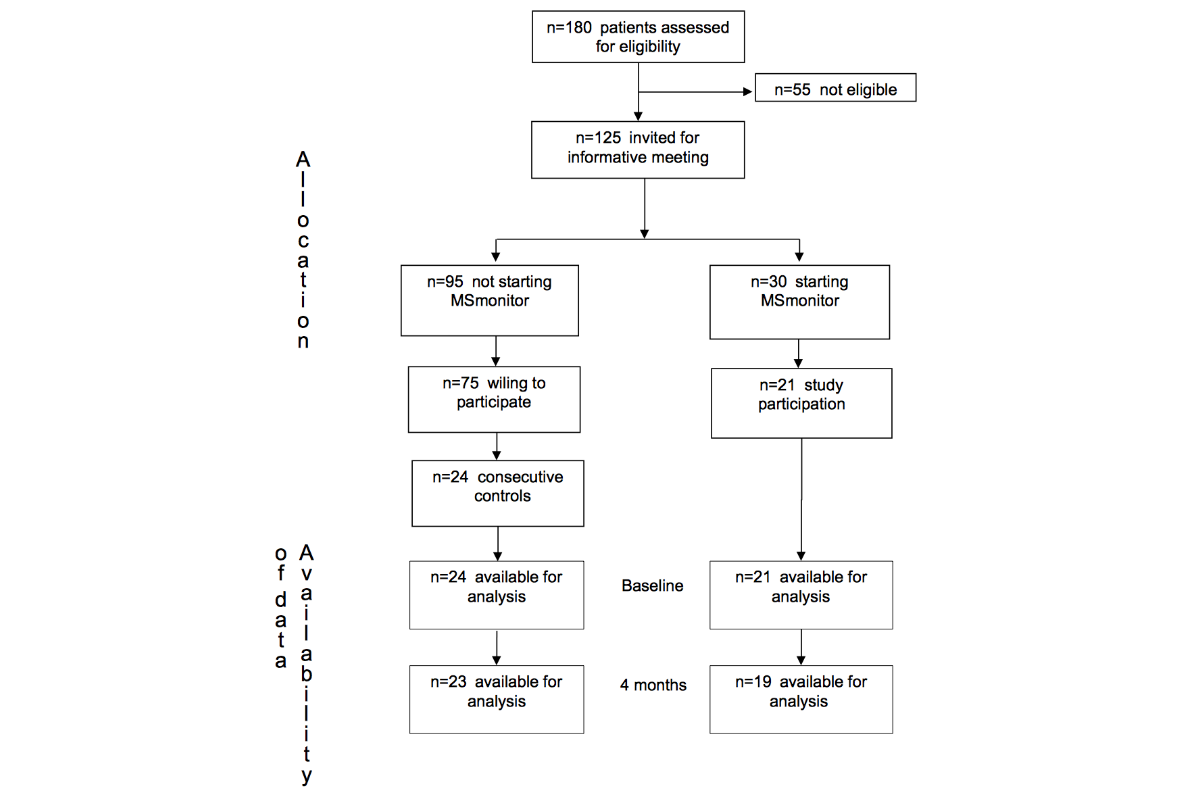
Study Flow Chart.

All patients registered with the diagnosis of MS constituted the study population (n=180; Figure 3).

The exclusion criteria for participation were as follows: diagnosis of clinically isolated syndrome, actual medical doubts about MS diagnosis, serious cognitive impairment, limited knowledge of the Dutch language, and nursing home residents. As a result, 55 patients were not eligible. The remaining 125 eligible patients were invited for a general meeting to be informed about MSmonitor and the study, and patients who had not attended the meeting were informed by phone. A total of 30 patients decided to start with the program and 21 of these were willing to participate in the study. Of the 95 patients who decided not to start with the program, 75 were willing to participate in the study, and out of those, 24 consecutive persons were recruited to form the control group. So, it was actually the patients who decided which study group to join (MSmonitor or control), and this fact explains the quasi-experimental design of the study.

### Ethical Approval and Informed Consent

The study did not qualify for being reviewed according to the Dutch Medical Research Involving Human Subjects Act of 1999. The study was carried out in compliance with the Declaration of Helsinki (Ethical Principles for Medical Research Involving Human Subjects version 2013; 64th World Medical Association General Assembly, Fortaleza, Brazil, October 2013) and the Dutch Medical Research Involving Human Subjects Act of 1999. Patients received no financial incentive or reward to participate. The study was approved by the hospital’s coordinator of local evaluation of medical experiments. Patients who agreed to participate signed an informed consent form.

### Data Collection

Patient-reported data were obtained by the use of paper-and-pencil questionnaires sent by regular mail, 1 week before the start of study participation and at follow-up. The questionnaires were accompanied by a stamped return envelope addressed to the neurological outpatient department for the attention of the principal researcher. Owing to financial restrictions, it was not feasible to provide e-versions of the questionnaires and to integrate these into the program. Data on MSmonitor utilization were provided by Curavista bv, Geertruidenberg, the Netherlands.

The following aspects of empowerment were assessed using psychometrically validated questionnaires: self-efficacy, participation, autonomy, and self-management (Table 2). We did not use a general or disease-specific empowerment measure, as such measures were not available in Dutch.

**Table 2:** Questionnaires used to assess aspects of empowerment.

Name	Purpose	Structure	Min^a^-max^b^	Validation
MSSES^c^ control	Confidence with managing symptoms and coping with demands of illness	9 items (10-100)	90-900 (higher=more confidence)	Schwartz et al [37] (MS^d^)
MSSES function	Confidence with regard to functional abilities	9 items (10-100)	90-900 (higher=more confidence)	Schwartz et al [37] (MS)
IPA^e^ limitations	Limitations to participation and autonomy	32 items (0-4)	0-128 (lower=less limitations)	Vazirinejad et al [38] (MS); Karhula et al [39] (MS)
IPA problems	Problems with limitations to participation and autonomy	9 items (0-2)	0-18 (lower=less problems)	Vazirinejad et al [38] (MS); Karhula et al [39] (MS)
PIH^f^ coping	Coping	3 items (0-8)	0-24 (lower=better coping)	Petkov et al [40] (CCC)^g^; Lenferink et al [41] (COPD^h^)
PIH symptoms	Recognition and management of symptoms	3 items (0-8)	0-24 (lower=better management of symptoms)	Petkov et al [40] (CCC); Lenferink et al [41] (COPD)
PIH adherence	Adherence to treatment	2 items (0-8)	0-16 (lower=better adherence to treatment)	Petkov et al [40] (CCC); Lenferink et al [41] (COPD)
PIH knowledge	Knowledge	4 items (0-8)	0-32 (lower=better knowledge)	Petkov et al [40] (CCC); Lenferink et al [41] (COPD)

^a^Min: minimum score.

^b^Max: maximum score.

^c^MSSES: Multiple Sclerosis Self-Efficacy Scale.

^d^MS: multiple sclerosis.

^e^IPA: Impact on Participation and Autonomy.

^f^PIH: Partners In Health.

^g^CCC: comorbid chronic conditions not including MS.

^h^COPD: chronic obstructive pulmonary disease.

Self-efficacy was assessed by the Multiple Sclerosis Self-Efficacy Scale (MSSES) [37], participation and autonomy were assessed by the Impact on Participation and Autonomy (IPA) questionnaire [38,39,42-44], and self-management behaviors and knowledge were assessed by the revised 12-item Partners In Health (PIH) scale [40,41].

At baseline, the level of education, degree of computer use, and degree of computer skills were assessed via multiple-choice questions (Table 3), as these factors may conceivably influence the speed with which persons become familiar with Web-based programs.

**Table 3:** Demographics, disease characteristics, level of education, degree of computer use, and degree of computer skills in the MSmonitor group and the control group.

Patient characteristics		MSmonitor (n=21)	Control (n=24)	*P* value
Female, n (%)		17 (81)	17 (71)	.43
Age (years), mean (SD)		45.4 (10.2)	49.3 (11.4)	.23
Disease duration (years), mean (SD)		8.7 (6.4)	12.2 (9.7)	.17
**Disease course, n (%)**				**.40**
	Relapsing remitting	16 (76)	13 (54)	
	Secondary progressive	4 (19)	7 (29)	
	Primary progressive	1 (4)	3 (12)	
	Benign	0 (0)	1 (4)	
**Education, n (%)**				**.39**
	Lower	6 (28)	10 (41)	
	Middle	12 (57)	7 (29)	
	Higher	3 (14)	7 (29)	
**Computer use, n (%)**				**.83**
	Several times per day	5 (23)	5 (20)	
	Daily	9 (42)	10 (41)	
	Several times per week	3 (14)	5 (20)	
	Once per week	0 (0)	1 (4)	
	Rarely or never	4 (19)	3 (12)	
**Computer skills, n (%)**				**.71**
	Rapidly familiar with new programs	7 (33)	7 (29)	
	Familiar after some log-ins	8 (38)	11 (46)	
	Difficulties with getting familiar	5 (24)	6 (25)	
	Impossible to get familiar	1 (5)	0 (0)	

### Data Analysis

For all outcomes, the absolute values at baseline and follow-up are presented as mean, standard deviation (SD), minimum, and maximum. As the study’s purpose was to investigate whether short-term changes could be observed after the start of MSmonitor usage, we compared in each group the follow-up with the baseline values by using multiple paired *t* tests. The baseline characteristics in the MSmonitor and control groups were tested for differences using *t* tests and χ² tests. The analyses were performed at the Department for Health Evidence, Radboud University Medical Centre, Nijmegen, the Netherlands. For all tests, a *P* value of <.05 was considered significant.

## Results

### Patient Characteristics

A total of 45 patients were included, 21 in the MSmonitor group and 24 in the control group. Demographics, characteristics of disease, level of education, degree of computer use, and degree of computed skills in both groups are presented in Table 3. There were no statistically significant differences with respect to gender, age, duration of disease, course of disease, level of education, degree of computer use, or degree of computer skills.

A total of 2 patients in the MSmonitor group and 1 in the control group failed to complete the follow-up questionnaires. Hence, the data analysis set comprised 19 MSmonitor and 23 control patients.

### MSmonitor Utilization

At 1 month, all 19 patients had used the Quick Scan. As not all patients started usage immediately after baseline assessment, the second and third Quick Scans were available to 11 and 7 patients, respectively, and these were used by 9 and 7 of them, respectively. Accordingly, the Quick Scan utilization rate was 95% (35/37). The MSIP was used by 14 out of 19 patients, and the MSQoL-54 by 4 out of 5 patients to whom it was available. So, in total, the Quick Scan, MSIP, and MSQoL-54 were available 61 times and were used 53 times, resulting in a combined completion rate of 87% for these 3 components. The Activity Diary was used by 13 (68%) patients; and in these, the mean (minimum, maximum) number of days of usage was 12 (1, 44). In all, the completion frequencies of Quick Scan, MSIP, and MSQoL-54, and the percentage of patients using the Activities Diary, resulted in an overall utilization rate of 83% of the MSmonitor components.

The Medication and Adherence Inventory part of the first Quick Scan showed that 10 of the 19 patients used a DMD and that 2 patients had missed 1 and 2 doses, respectively, in the preceding month. Moreover, all patients completed the first Quick Scan, whereas patients who also used the Activity Diary completed the second Quick Scan more frequently than those who did not use the Activity Diary (8/11 vs 1/8).

### Empowerment Outcomes

The mean, SD, minimum, and maximum values of the various outcome scores at baseline and at follow-up are presented in Table 4.

In the MSmonitor group, scores remained unchanged for MSSES control, MSSES function, IPA limitations, IPA problems, PIH coping, PIH recognition and management of symptoms, and PIH adherence to treatment. The mean PIH knowledge score decreased, suggesting an improvement. In the control group, all scores were unchanged.

**Table 4:** Mean (SD) and minimum-maximum values of Multiple Sclerosis Self-Efficacy Scale, Impact on Participation and Autonomy, and Partners In Health scores at baseline and at follow-up in the MSmonitor and control groups.

Empowerment aspects	MSmonitor (n=19)			Control (n=23)		
	Baseline, mean (SD); minimum-maximum	4 months, mean (SD); minimum-maximum	*P* value	Baseline, mean (SD); minimum-maximum	4 months, mean (SD); minimum-maximum	*P* value
MSSES^a^ control	59.8 (19.0), 20.0-87.8	63.4 (17.7), 32.2-94.4	.19	55.3 (19.5), 25.6-96.7	53.7 (20.8), 22.2-91.1	.40
MSSES function	72.4 (22.4), 28.9-100	73.5 (21.8), 30.0-100	.62	67.8 (24.3), 21.3-100	66.5 (23.5), 26.7-100	.17
IPA^b^ limitations	2.8 (0.4), 1.7-3.6	2.7 (0.6), 1.6-3.7	.26	2.6 (0.7), 1.1-4.0	2.6 (0.6), 1.5-3.95	.28
IPA problems	0.71 (0.36), 0.00-1.71	0.79 (0.48), 0.00-1.86	.40	0.97 (0.35), 0.22-1.71	0.85 (0.35), 0.00-1.43	.25
PIH^c^ coping	19.2 (3.3), 13.0-24.0	18.9 (4.8), 4.0-24.0	.73	18.6 (3.9), 8.0-24.0	18.9 (3.2), 12.0-24	.76
PIH symptoms	21.9 (1.2), 20.0-24.0	21.6 (2.3), 16.0-24.0	.52	20.8 (2.2), 16.0-24.0	20.9 (2.6), 13.0-24	.87
PIH adherence	15.1 (1.4), 11.0-16.0	14.9 (2.2), 8.0-16.0	.80	14.8 (1.4), 12.0-16.0	14.6 (2.3), 7.0-16	.34
PIH knowledge	28.7 (2.0), 25.0-32.0	27.8 (1.7), 24.0-30.0	.02	27.7 (3.4), 21.0-31.0	28.7 (2.2), 24.0-32	.24

^a^MSSES: Multiple Sclerosis Self-Efficacy Scale.

^b^IPA: Impact on Participation and Autonomy.

^c^PIH: Partners In Health.

## Discussion

### Principal Findings

We conducted a quasi-experimental study in first-time users of MSmonitor to explore the program’s early effects on empowerment and found that at 4 months’ follow-up, self-efficacy, participation, autonomy, and self-management did not change, whereas knowledge had increased. The increase in the PIH knowledge score was about 0.9 SD baseline, which suggests that the change was clinically meaningful and can therefore be qualified as an improvement. It is, however, not sure to what degree the better knowledge results from the utilization of MSmonitor because at the time of the study, the program had not included an information function with links to websites of patient organizations and health care organizations.

In all, our findings suggest that early improvement of patient empowerment is unlikely to occur after starting MSmonitor. This result is clinically relevant as it may be communicated to first-time users to prevent them from having unrealistic expectations about the program’s effects. Similarly, health care professionals should not expect their patients to have a better control of their situation, increased participation in care processes, or improved self-management in the short term [3,8,9]. A lack of utilization is unlikely to explain the unchanged empowerment outcomes, as the program’s utilization was high; the overall rate being 83%.

An additional finding was the association between the completion of the Quick Scan and the use of the Activity Diary. Although all patients completed the first Quick Scan, those who also used the Activity Diary evidently completed the second Quick Scan more frequently. This suggests a substantial relationship between repeated self-assessments of fatigue and HRQoL on the one hand and the documentation of activities and resting periods on the other hand and is in agreement with the hypothesis on the role of the program in self-management of fatigue. It may therefore be promising for future research on the effects of MSmonitor to focus on medium- to long-term changes in fatigue and on how patients self-manage their fatigue.

### Limitations

The study has several limitations. First, the sample size, and therefore the chance of achieving statistically significant results, was rather low. Nevertheless, it may have been large enough to detect clinically relevant changes, as is suggested by the improved PIH knowledge score. Second, the study group was heterogeneous. Self-management perspectives and goals may differ between relapsing remitting, secondary progressive, and primary progressive patients. Moreover, the failure to detect a change in the PIH adherence to treatment score may relate to the fact that 9 of the 19 patients were not treated with a DMD, whereas only 2 of the DMD-treated patients reported (a low number of) missed doses [45]. Actually, the low incidence of DMD treatment in our patients may be explained by the fact that 1 out of 4 had progressive MS, and that in general 1 out of 4 patients with relapsing-remitting MS is not treated with a DMD [45]. Third, we included 5 patients in the MSmonitor group with reportedly low computer skills and 1 patient with reportedly no such skills, without offering them further training or education. This may have prevented these patients from optimally using the program. Moreover, owing to financial restrictions, the technology was introduced to patients and health care professionals in a single introductory session, without further staff training or education of patients; the absence of an optimal embedding in the daily life of patients and practices of health care providers may have negatively influenced the occurrence of short-term effects. Fourth, patient involvement in the developing process of MSmonitor consisted of receiving patient feedback on a continuous basis via the program’s helpdesk and by means of meetings in the hospitals where the program was implemented. However, there were no cocreation sessions or focus group meetings, and this may be considered a limitation. Fifth, the study was not randomized. The fact that it was the participants who decided which group to join may have biased their reporting. As, however, both groups failed to show changes at follow-up (except for knowledge in the MSmonitor group), this limitation seems of minor importance. Finally, some aspects of empowerment were not covered by the questionnaires, such as support and patient-provider interaction [46,47].

### Comparison With Prior Work

Few studies have investigated the effect of Web-based self-management and care programs on empowerment in patients with MS, and the results are ambiguous. In a 6-month uncontrolled study (n=31) on the perceived benefits of Web-based MS-related patient-reported outcome collection, nearly 52% of the participants reported improved understanding of their disease [48]. Similarly, in a survey among MSmonitor users (n=55), 46% reported that their insight into symptoms and disabilities had increased since the use of the program [24]. On the other hand, in a 12-month randomized controlled trial (n=206), the expansion of an electronic MS health record with a self-monitoring and self-management system did not result in improved self-efficacy or symptoms [13]. Combined with the findings of this 4-month quasi-experimental study, the available data suggest that patient empowerment is not necessarily affected by the use of MS-related Web-based self-management and care programs.

Notably, self-management is a major issue for patients with MS. A recent study showed that positive expectations about the helpfulness for self-management is an important predictor for the acceptance of MS-related apps [49]. Actually, 26% of the available MS-related apps have been designed for self-management purposes [50]. On the other hand, a recent review showed that the available MS apps fail to sufficiently meet the needs and demands of patients [51]. Although education and personal data management were the frequently included features, remote monitoring and fatigue management were often not present [51], despite the fact that fatigue management functions in mobile health solutions are important to patients with MS [52]. So, it seems that because of its fatigue management, monitoring and—recently added—information functions, MSmonitor compares favorably with the majority of MS apps [52].

The program’s overall utilization rate was 83%, whereas in a previous survey among all MSmonitor users, the most frequently used components Medication and Adherence Inventory, Activity Diary, and MSIP were used by 55%, 47%, and 40% of the respondents, respectively [23]. It is known that long-term Web-based self-monitoring in patients with MS is hampered by a declining adherence, both in regular care and in direct-to-patient research settings [48,53]. Thus, in a 6-month study (n=31) with monthly completion of 5 questionnaires, it was found that all questionnaires were completed less frequently in the second 3 months [48]. Interestingly, a recent study suggests that continuous communication with patients may promote the continued use of digital data collection tools [54]. Moreover, adaptation of digital self-monitoring tools to patients’ personal situation, giving guidance to increase the value of their data, and integration of digital self-monitoring into treatment plans might also increase the adherence of patients with MS to Web-based programs and apps [55].

Finally, a recent review identified over 100 MS-related apps, but in none was evidence found in the literature on evaluation of the effects [50]. This may be worrisome, as the widespread implementation and utilization of MS-related Web-based programs, including mobile apps, will most likely depend on whether convincing evidence can be obtained regarding their effectiveness and cost-effectiveness [50]. Owing to limited resources, it is unlikely that all available tools will be evaluated in randomized controlled trials [56]. Moreover, the external validity of trial results is not self-evident, given that patient preferences may differ between regions or countries and preferences may change over time and the ongoing development of the tools. Therefore, prospective observational studies in real-world settings and retrospective studies using large databases are increasingly being considered as alternatives for obtaining actionable data [56,57].

### Conclusions

In a quasi-experimental study, we investigated short-term changes in empowerment in patients with MS who started using the Web-based program MSmonitor. At 4 months, self-efficacy, participation, autonomy, coping, recognition and management of symptoms, and adherence to treatment did not change. The utilization rate of the program’s components was high. Our findings suggest that it may not be justified for first-time users of MSmonitor and their health care providers to expect a short-term improvement in empowerment. Immediate effects might be realized by better informing patients about the option to give the multidisciplinary team access to their data, as this may influence treatment decisions and care at short notice. It may well be that the program becomes effective in the medium to long term because of patients becoming increasingly familiar with the various components and their possibilities. A better adjustment of the program to the expectations and wishes of patients—in terms of content, personalization, and integration into treatment plans—is expected to also enhance empowerment [55].

## Abbreviations

DMD: disease-modifying drug
HADS: Hospital Anxiety and Depression Scale
HRQoL: health-related quality of life
IPA: Impact on Participation and Autonomy
LMSQoL: Leeds Multiple Sclerosis Quality of Life
MFIS-5: Modified Fatigue Impact Scale-5 items
MS: multiple sclerosis
MSIP: Multiple Sclerosis Impact Profile
MSQoL-54: Multiple Sclerosis Quality of Life-54
MSSES: Multiple Sclerosis Self-Efficacy Scale
PIH: Partners In Health
